# Quality Improvement of Garlic Paste by Whey Protein Isolate Combined with High Hydrostatic Pressure Treatment

**DOI:** 10.3390/foods12071500

**Published:** 2023-04-03

**Authors:** Baoyuan Zang, Zhichang Qiu, Zhenjia Zheng, Bin Zhang, Xuguang Qiao

**Affiliations:** Key Laboratory of Food Processing Technology and Quality Control of Shandong Higher Education Institutes, College of Food Science and Engineering, Shandong Agricultural University, 61 Daizong Street, Tai’an 271018, Chinaxgqiao@sdau.edu.cn (X.Q.)

**Keywords:** whey protein isolate, high hydrostatic pressure, garlic paste, green discoloration, volatile compounds

## Abstract

Garlic, one of the most popular spices and medical herbs, has a unique pungent flavor and taste. Conventional homogenization and thermal treatment commonly lead to flavor and color deterioration in garlic paste, because allicin is highly susceptible to degradation and reaction. The present study was to investigate the effects of whey protein isolate (WPI) and different levels of high hydrostatic pressure (HHP, 200, 300, 400, 500, and 600 MPa) on the quality of garlic paste. Results showed that the addition of WPI in the homogenization of garlic significantly prevented green discoloration. Furthermore, WPI plus HHP under 500 MPa could better protect the color of garlic paste. Higher pressure (600 MPa) led to WPI aggregation, resulting in higher green color chroma of garlic paste. GC-MS results revealed that the application of WPI and HHP in garlic paste increased the relative level of pungent flavor compounds and decreased those of unpleasant odor compounds. The correlation analysis results revealed that WPI efficiently prevented garlic green discoloration, which is attributed to the thiol group in WPI exchanging the sulfonyl groups in allicin. In consideration of the microbial load, flavor and color quality of garlic paste, the optimal processing conditions were found at 500 MPa for 5 min with 2% WPI addition, extending shelf life to 25 days.

## 1. Introduction

Garlic (*Allium sativum* L.) contains abundant nutritional components (e.g., polysaccharides, proteins, lipids and organosulfur compounds) and has been widely used as a seasoning or herbal medicine around the world [1,2]. Previous studies have shown that the typical flavor of fresh garlic and numerous bioactive activities of garlic are mainly attributed to the organosulfur compounds [3]. Among them, allicin is the predominant constituent of organosulfur compounds (accounting for 70–80%) and, therefore, can be used as an indicator of the quality of garlic products [4]. However, allicin is unstable and easily decomposes into other, secondary sulfur-containing components, leading to flavor deterioration. In minced, crushed, sliced, pureed, or other mechanical disruption of garlic, intensely green pigments are often formed during processing, which represents the discoloration from a cream white to green color [5]. Allicin also plays a key role in garlic green discoloration. These problems of flavor deterioration and green discoloration greatly limit the development and consumption of processed garlic products [6].

In order to improve the stability of flavor and inhibit the discoloration of garlic products, some measures have been applied to garlic processing, such as blanching and the addition of chemical additives, which are applied to the intact garlic to kill alliinase [7]. Although the garlic products treated with thermal processing and pH adjustment can maintain good appearance, their pungent flavor and biological activities are completely lost [8,9]. Compared with thermal processing, high hydrostatic pressure (HHP) treatment minimized this effect on the physicochemical properties and biological activities of garlic products, but green discoloration still occurred [10,11]. The conjugation of allicin and cysteine was reported to enhance the stability of allicin, which was beneficial to its antioxidant and anticancer activities [12,13,14]. This is mainly due to the exchange of the thiol group in cysteine with the sulfonyl groups in allicin to produce stable disulfide bonds. However, the amount of cysteine is strictly limited as a food additive. For example, the maximum addition in flour-based foods must be less than 0.06 g/kg [15]. The excessive addition of cysteine may pose considerable risks in food. Considering that the reaction of thiol groups and sulfonyl groups can occur between allicin and proteins with free sulfonyl groups, proteins have high potential to stabilize allicin. Jiang et al. [15] reported that ultrasound-assisted binding of allicin and whey protein isolates exhibited better stability and emulsification. Huang et al. [16] found that allicin–soy protein isolate conjugates not only caused structural changes in the protein, but also increased the thermal stability and antioxidant activity of allicin. Therefore, it is of interest to investigate the effect of natural protein isolates on the quality of garlic products. Previous studies generally focused on the physiochemical properties and bioactivity of allicin–protein conjugates. To our knowledge, there are no studies that have reported the application of proteins in garlic products.

Whey protein isolate (WPI) is recognized as a high-quality animal protein because of its high digestion/absorption properties and great nutritional value [17]. In this study, the effect of WPI addition and HHP treatment on microbial load, color characteristics and allicin content in garlic paste were investigated. The results were used to prevent flavor deterioration and green discoloration of garlic paste. The flavor profiles of HPP-WPI treated garlic paste were evaluated by solid-phase microextraction-gas chromatography-mass spectrometry (SPME-GC-MS). The results revealed the interaction effects of WPI and HHP treatment on garlic quality and provided a valuable strategy for the improvement and quality control of garlic products.

## 2. Materials and Methods

### 2.1. Materials and Reagents

Garlic was purchased from Laiwu (Shandong, China). Diallyl disulfide (>98% purity, HPLC grade) was obtained from Sigma-Aldrich Co., Ltd. (Shanghai, China). Ascorbic acid (>99% purity) was purchased from Hualan Chemical Technology Co., Ltd. (Shanghai, China). Whey protein isolate (90% purity) was obtained from Maclin Co., Ltd. (Shanghai, China). Acetonitrile and methanol (HPLC grade) were purchased from Yuwang Chemical Co., Ltd. (Dezhou, China). All other chemicals were of analytical grade.

### 2.2. Preparation of Garlic Paste

The garlic paste was prepared according to the procedure of Jiang et al. [15], with some modifications. Briefly, a 2% WPI solution was sonicated (40 kHz, 25 W/L) at 60 °C for 15 min to increase the exposure of sulfhydryl groups. Then, peeled garlic was homogenized with 2% WPI (1:2, *m*/*v*) to obtain garlic paste. The pH of garlic paste was adjusted to 4.0 with 0.04 g/mL ascorbic acid solution. The final concentration of ascorbic acid in garlic paste was about 2.50 mg/g. The resulting garlic paste was packed (50 g for each pouch) and sealed, followed by HPP treatment at 200–600 MPa for 5–25 min (operated at 25 °C) to investigate its effect on the quality of the garlic paste. The HHP equipment (Bao Tou KeFa High Pressure Technology Co., Ltd., Baotou Inner Mongolia, China) was equipped with a reservoir volume of 3 L, and the pressure generated within the chamber was automatically adjusted by a digital computer. The garlic paste was freshly prepared using purified water to homogenize (pH 5.77), then treated with pH adjustment and HHP according to the above procedure to obtain the original garlic paste (GP) and HHP-GP.

### 2.3. Microbiological Analysis

As described in a previous report [6], 15 g garlic paste was homogenized with 135 mL sterile saline (8.5 g/L NaCl). The obtained sample was serially diluted 10-fold with sterile saline. Subsequently, 0.5 mL of diluent was fully mixed with 20 mL plate count agar medium in a sterile Petri dish and incubated upside-down at 36 °C for 48 h. The colony count was calculated and expressed as Log CFU/g.

### 2.4. Color Index Analysis

The color index of garlic paste was evaluated using a CR-10 Plus colorimeter (Kefeng Instrument Co., Ltd., Baoding, Hebei, China). The different garlic paste products were placed in Petri dishes for 6 h and directly measured at 25 °C. The L* value represents lightness, a* the degree of red (a* > 0) or green (a* < 0), and b* the degree of yellow (b* > 0) or blue (b* < 0). Saturation (chroma, C*) is used to represent the purity of the color, and color difference (Δ*E*) is used to represent the overall color change. C* and Δ*E* were calculated according to the following formulas:

(1) $$C^{*}=\sqrt{a^{*2}+b^{*2}}$$

(2) $$\Delta E^{*}=\sqrt{{\left(\Delta L^{*}\right)}^{2}+{\left(\Delta a^{*}\right)}^{2}+{\left(\Delta b^{*}\right)}^{2}}$$

where ΔL*, Δa* and Δb* indicates the color difference between the color indices of the processed garlic paste and the original garlic paste.

### 2.5. Allicin Content Analysis

The extraction and analysis of allicin was carried out according to Zhang et al. [18], as based on Tocmo’s method [19]. Briefly, an equal volume (*v/w*) of n-hexane was added to 10.0 g garlic paste to extract allicin (3 times). The organic phases were combined and concentrated using an air stream at 25 °C. The final residue was dissolved in 60% methanol, filtered, and analyzed using high performance liquid chromatography (HPLC). Allicin content analysis was performed on a Shimadzu LC-20AT system (Kyoto, Japan) equipped with an InertSustain C18 (4.6 × 9 × 250 mm, 5 μm) column. The mobile phase was acetonitrile: water: methanol (50:41:9), and the flow rate was at 1.0 mL/min. To accurately determine allicin content, allicin external standards were synthesized and isolated according to our previous report [18].

### 2.6. Analysis of Free Sulfhydryl (SH) Content

The SH content was determined according to Ayim et al. [20] with some modification. Briefly, garlic paste was diluted with distilled water (1:5, *m*/*v*) to obtain garlic extract. Next, 0.5 mL garlic extract was mixed with 5 mL Tris-Gly buffer, then 20 µL Ellman’s reagent was added. The final mixture was reacted at 25 °C for 30 min. After that, the absorbance was measured at 412 nm using a spectrophotometer (BioTek Instruments, Charlotte, VT, USA). A blank group was prepared using buffer instead of garlic extract. The SH group contents were calculated using Equation (3) [15]:

(3) $$C_{SH}\;\left(\mu \mathrm{mol}/g\right)=\left(73.53\times \Delta A_{412}\times D\right)/C$$

where D represents dilution coefficient, *C* represents the protein concentration in garlic extract (mg/mL), and Δ*A*_412_ is the differential between the treatment group and original garlic paste.

### 2.7. Analysis of Volatile Components

#### 2.7.1. Extraction of Volatile Components

Briefly, 2.0 g garlic paste was exactly weighed and transferred into a 10 mL headspace vial, followed by air-tight sealing with a Teflon-coated rubber septum and an aluminum cap. A 75 μm DVB/CAR/PDMS SPME fiber was first conditioned at 250 °C for 3 min. Then, headspace extraction of the volatile compounds was carried out with the SPME fiber at 50 °C for 30 min. After that, the SPME fiber was immediately transferred to the inlet of GC, and volatile components were desorbed at 250 °C for 10 min.

#### 2.7.2. GC-MS Analysis

GC-MS analysis was conducted following the method of Chen et al. [21] with some modifications on a Shimadzu TQ8030 GC–MS (Shimadzu Co., Ltd., Kyoto, Japan) coupled with an Agilent DB-5MS column (30.0 m × 0.25 mm i.d., 0.25 μm, Agilent Technologies, Santa Clara, CA). The oven temperature was initially held at 50 °C for 4 min, then increased to 65 °C (increase rate, 1 °C/min), 75 °C (increase rate, 10 °C/min), 90 °C (increase rate, 1 °C/min), 210 °C (increase rate, 3°C/min), and finally heated to 280 °C at a rate of 10 °C/min and held for 10 min. High-purity helium (99.99%) was used as the carrier gas at a fixed flow of 1.0 mL/min (split ratio, 1:30). The MS conditions were as follows: injector temperature of 280°C, ion source temperature of 230 °C, ionization voltage of 70 eV, scanning range of 30–450 *m*/*z*. The volatile compounds were identified using the NIST 11 mass spectrometry library. The characteristic flavor compounds in garlic (including diallyl sulfides and vinyl-dithiins) were identified by external standards prepared by our laboratory [18] and commercially available standards. The peak normalization method was used for calculating the relative abundance of each compound in the garlic paste.

### 2.8. Microbiological Stability

Garlic paste after processing (HHP-WP) was stored at 25 °C and 4 °C to determine its microbiological stability during storage. The colony count analysis was as described in Section 2.3. The SGompertz growth equation was used to describe microbial behavior and predict the shelf life of HHP-WP. The SGompertz model equation is derived from the Gompertz model, calculated using Equation (4):

(4) $$\left[Log\left(\frac{Nt}{N0}\right)\right]=a\times \exp\left\{-\exp\left[-k\left(t-M\right)\right]\right\}$$

where *N_t_* and *N*_0_ represent the colony count on day *t* and day 0, respectively; *a* represents the logarithmic value of the total number of bacterial colonies as time increases. *K* represents the relative maximum growth rate; and *M* represents the time required to obtain the maximum growth rate [22].

### 2.9. Statistical Analysis

The results were expressed as mean values ± standard deviation, analyzed using SPSS 26.0 (IBM Corporation, Armonk, NY, USA). One-way analysis of variance (ANOVA) and Duncan’s test (*p* < 0.05) were used to evaluate the statistical differences. All treatments were determined independently in triplicate.

## 3. Results

### 3.1. Effects of WPI and HHP Treatments on Microbial Load of Garlic Paste

**Figure 1:**
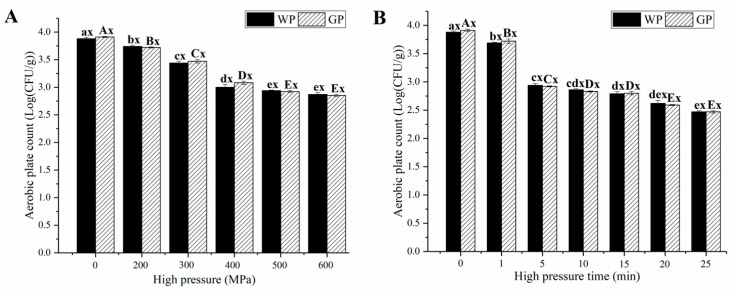
Effect of (**A**) HHP treatment and (**B**) pressure holding time at 500 MPa on microbial inactivation. “a–e” and “A–E” mean within WP and GP group, respectively, while different letters indicate a significant difference (*p* < 0.05). “x” means there was no significant difference (*p* ≥ 0.05) between WP and GP under the same HHP condition.

Figure 1 shows the effect of HHP pressure and time on the total number of microbial colonies. The total number of colonies in the original garlic paste was 3.88 ± 0.22 Log (CFU/g), which was similar to that of the garlic paste supplemented with WPI (WP) (*p* > 0.05). This indicated that there was no significant effect on the microbial load of the garlic paste due to the addition of WPI. As shown in Figure 1A, the number of colonies showed a significant (*p* < 0.05) decrease as the HHP pressure was increased from 0 MPa to 500 MPa and held for 5 min. Further increases in HHP pressure only resulted in a slight decrease in the number of colonies (*p* > 0.05). The response of microbial load to HHP time followed a similar trend with HHP pressure (Figure 1B). The most effective treatment for microbial inactivation was achieved at 500 MPa for 25 min, with a total number of colonies of 2.47 ± 0.02 Log CFU/g. At a longer HHP times (>5 min), the decrease in the number of colonies was slower, which might have been due to the fact that pressure was not uniformly transmitted throughout the garlic paste [23]. In order to obtain the limited microbial index (3.70 Log CFU/g), the garlic paste was treated by HHP at 500 MPa for at least 5 min.

### 3.2. Effects of WPI and HHP Treatments on Color Appearance

**Figure 2:**
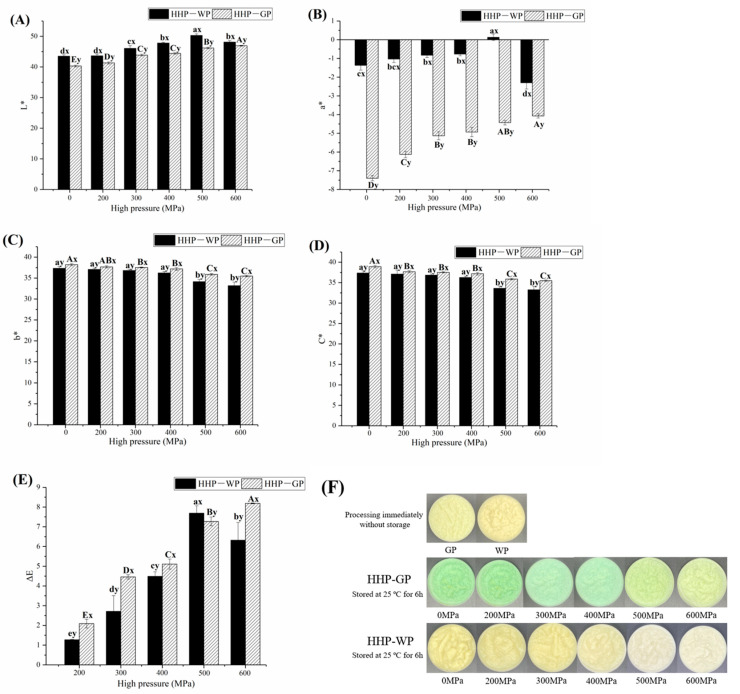
Effect of HHP treatment on garlic paste color indexes and appearances. (**A**) L*, (**B**) a*, (**C**) b*, (**D**) color chroma, C*, (**E**) total color difference, ΔE, (**F**) color appearance. “a–e” and “A–E” mean within HHP-WP and HHP-GP group, respectively, while different letters indicate a significant difference (*p* < 0.05). “x-y” with different letters indicates a significant difference (*p* ≥ 0.05) between HHP-WP and HHP-GP under the same HHP conditions.

The color parameters and appearance of original and processed garlic paste were measured after incubating at 25 °C for 6 h (Figure 2). Freshly homogenized garlic paste was cream white. In Figure 2, the original garlic paste shows an a* value of −7.4 ± 0.14 and C* value of 38.20 ± 0.24, with a vivid green color by visual observation (Figure 2F). In comparison, the a* value of WP was significantly increased to −1.37 ± 0.25 (*p* < 0.05) and appeared as a bright white appearance, which suggested that the treatment with WPI could effectively inhibit the greening of garlic paste. As the HHP pressure increased, the L*, a* and ΔE values of GP showed an increasing trend, while the b* and C* values gradually decreased, with significant differences between most of the different treatment groups (*p* < 0.05). As described in previous studies, the green discoloration began with a vivid green color, then the green color faded and turned to green-yellow [5,11]. Therefore, the above result indicated that HHP treatment effectively accelerated the green discoloration. Although the color parameters of WP had variation characteristics similar to those of GP at different HHP pressures, it usually had significantly lower b*, C* and ΔE values. Significantly higher L* and a* values were also observed at the same pressure between WP and GP (except for ΔE values at 500 MPa) (*p* < 0.05). Notably, the a* value of WP was only −2.3 ± 0.13, considering that the a* value had a higher chromaticity property that affected the color appearance [24]. The large visual difference in appearance can be seen in Figure 2F. These results indicate that WPI combined with HHP treatment can maintain the color of original garlic paste without storage. At 500 MPa, WP obtained the lowest a* and C* values and highest L* values, presenting the whitest appearance (Figure 2F). The decrease in green color and increase in brightness could be attributed to the inactivation of polyphenol oxidase at 200–500 MPa [25]. Notably, further increases in HHP pressure led to an increase in chromaticity (C*), which might have been caused by the occurrence of WPI aggregation at 600 MPa [26].

**Figure 3:**
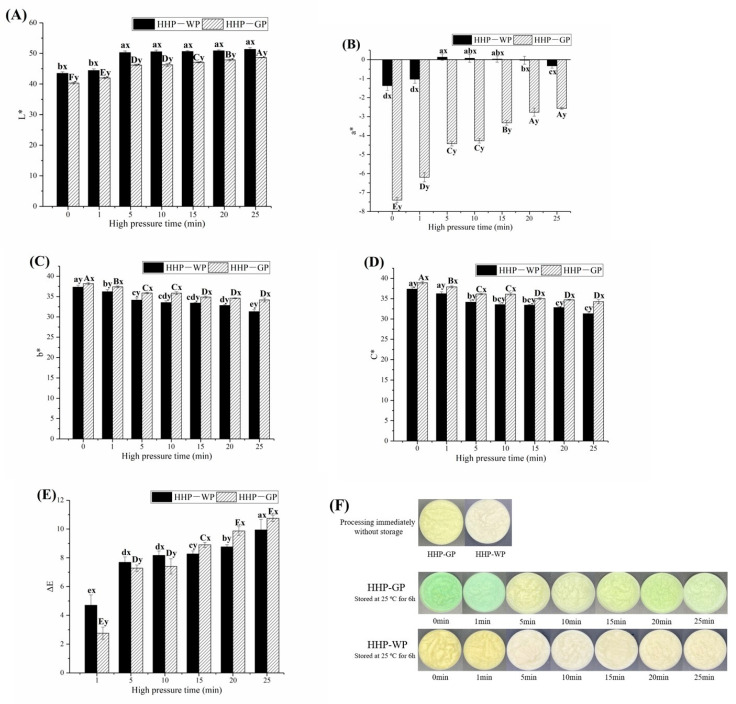
Effect of HHP time on garlic paste color indexes and appearances. (**A**) L*, (**B**) a*, (**C**) b*, (**D**) color chroma, C*, (**E**) total color difference, ΔE, (**F**) color appearance. “a−e” and “A−E” mean within HHP-WP and HHP-GP group, respectively, while different letters indicate a significant difference (*p* < 0.05). “x-y” with different letters indicates a significant difference (*p* ≥ 0.05) between HHP-WP and HHP-GP under the same HHP conditions.

As shown in Figure 3, for WP and GP treated with 500 MPa held for different treatment durations, the color parameters and visual appearance of garlic paste were monitored after incubating at 25 °C for 6 h. As the treatment time increased, the L*, a* and ΔE of WP and GP showed an increasing trend. Contrarily, both b* and C* decreased in WP and GP. For WP, the highest L* and a* values appeared at 20 min, and extending the processing time to 25 min resulted in a decrease in a* and C*, corresponding to light green color appearance in HHP-WP at 25 min (Figure 3F). This result suggests that HPP processing time above 25 min at 500 MPa might lead to the aggregation of WPI and be detrimental to the maintenance of the color of WP.

### 3.3. Effects of WPI and HHP Treatments on Allicin Content and Thiol Groups

**Figure 4:**
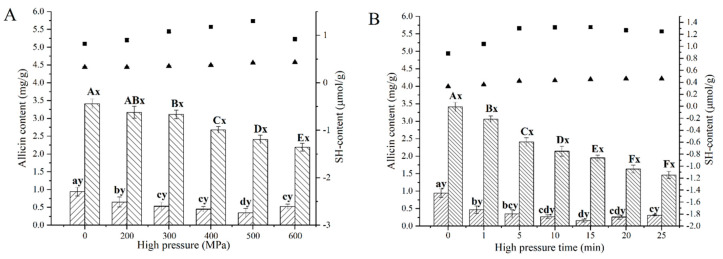
Effect of (**A**) HHP and (**B**) pressure holding time at 500 MPa on the contents of allicin and thiol groups. 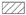 represents allicin content in HHP-WP; 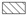 represents allicin content in HHP-GP; ■ represents the content of SH groups in HHP-WP; ▲ presents the content of SH groups in HHP-GP. “a–d” and “A–F” mean within HHP-WP and HHP-GP group, respectively, while different letters indicate a significant difference (*p* < 0.05). “x-y” with different letters indicates a significant difference (*p* ≥ 0.05) between HHP-WP and HHP-GP under the same HHP conditions.

The effects of WPI combined with different HHP levels on the content of allicin and free thiol (SH) groups are shown in Figure 4A. As shown in Figure 4A, in raw garlic, the allicin content was 3.41 ± 0.13 mg g^−1^. After treating with WPI, the allicin content was sharply reduced, to 0.94 ± 0.12 mg g^−1^. This result indicated that allicin reacted with the thiol groups in WPI [15]. With the increase in pressure, the allicin content in HHP-GP decreased continuously from 3.41–2.19 mg g^−1^, whereas the SH groups increased continuously from 0.33–0.43 μmol g^−1^ (Figure 4A). It could be explained that HHP promotes green discoloration, in which allicin participated [11,27], and the increase in thiol groups was attributed to the ability of allicin to cleave disulfide under high pressure [28]. In HHP-WP, the minimal allicin content of 0.35 mg g^−1^ was achieved at 500 MPa, with a maximal SH content of 1.3 μmol g^−1^. Carullo et al. [26] reported that 200–400 MPa HHP treatment contributed to trigger partial unfolding of WPI, resulting in unmasking of buried thiol groups. More severe HHP treatment (600 MPa) is commonly accompanied by the occurrence of protein aggregation, which leads to a decrease in free thiol groups [25,29]. Therefore, more allicin was used to bind WPI at 200–500 MPa, decreasing allicin content. In addition, the increase in allicin at 600 MPa might due to the reversibility of allicin binding to the residues, which was in agreement with allyl isothiocyanate binding β-lactoglobulin [30].

The changes in allicin content and thiol groups in HHP-WP under 500 MPa over processing time are shown in Figure 4B. With increasing HHP processing time, the allicin content decreased first, then increased, and the SH content showed a contrary trend of change. This indicated that longer processing time at 500 MPa also leads to WPI aggregation. The higher allicin content was achieved at 5, 10, and 15 min, corresponding 1.30, 1.32, and 1.32 mg g^−1^, respectively, showing no significant difference (*p* > 0.05).

Based on the above results for microbial inactivation, color appearance and allicin content, additional investigations on the influence of WPI combined with HHP treatment on the flavor compounds in garlic paste, in comparison with untreated and only WPI treated samples, were performed, setting HHP parameters at 500 MPa for 5 min.

### 3.4. Effects of WPI and HHP Treatments on Flavor Compounds

SPME–GC–MS was conducted to study the influence of WPI and HHP treatments on the sensory aroma of garlic paste. A total of 38 compounds were identified in original garlic paste, as described in our previous report [6], and 41 components were observed in the WP. After HHP treatment, another new component was generated in HHP-WP. Moreover, the signal intensities of individual compounds among three samples showed significant differences, indicating that the aroma compounds were increased, decreased, or generated due to addition of WPI and HHP treatment.

As shown in Table 1, in original garlic paste, the highest relatively abundant volatile flavor component was diallyl disulfide (DADS, 19.36%), followed by diallyl trisulfide (DATS, 16.57%), 3-Vinyl-3,4-dihydro-1,2-dithiin (3-VDT, 11.45%), and 2-Vinyl-2,4-dihydro-1,3-Dithiin (2-VDT, 8.60%). Our results were in agreement with those of Ferioli et al. [31]. These organosulfur compounds were related to the typical garlic scent, garlic pungency and odor, as reported by previous studies [6,19,32]. Those predominant volatile compounds, linear polysulfides (DADS and DATS), have strong volatilities with a pungent smell [33]. It was reported that 2-VDT and 3-VDT were the major transformation products of allicin in GC analysis [19]. The dominant volatile compounds in WP and HHP-WP were similar to those in original garlic paste; however, the relative proportions showed significant differences. In WP and HHP-WP, DADS was increased to 23.50% and 28.91%, respectively. This result indicated that WPI combined with HHP treatment could maintain, even increase, the pungent smell of garlic. The 2-VDT and 3-VDT continuously decreased to 4.17% and 5.96%, respectively, after WPI combined with HHP treatment, which corresponded to a decrease in allicin. 

The application of WPI combined with HHP treatment resulted in a significant decrease (*p* < 0.05) in methallyl disulfide, and a relative decrease in methallyl sulfide. These compounds were commonly recognized as breath metabolites of allicin with unpleasant smell [34]. Moreover, cyclotrisiloxane hexamethyl was observed in WP and HHP-WP and identified as the main bioactive compound in Dillenia scabrella leaves [35]. Additionally, 2,4-dimethyl-thiophene (0.244%) was generated during HHP treatment. Several thiophenes were identified in pyrolysis bio-oils, which were cyclic structures generated through free radical reactions by smaller sulfur compounds [36], recognized as the most stable sulfur-containing compounds. Thus, it is not surprising that the dimethyl thiophene were determined under high HHP.

In summary, WPI combined with HHP treatment increased the levels of the predominant volatile aroma components and decreased unpleasant odor. Additionally, the presence of four new compounds gave HHP-WP a richer and balanced odor.

### 3.5. Changes in the Microbial Count of Garlic Paste during Storage

**Figure 5:**
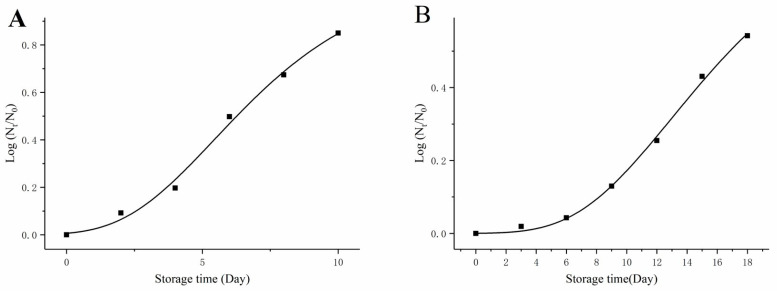
Changes in the number of colonies in HHP-WP during storage at 25 °C (**A**) and 4 °C (**B**). The scatter plots represent the number of colonies at different times, and the curve represents the fitting of the SGompertz model.

The total number of colonies in freshly homogenized garlic paste was 3.91 ± 0.23 Log CFU/g, and 2.94 ± 0.17 Log CFU/g in garlic paste after WPI-HHP (500 MPa, 5 min) treatment. As shown in Figure 5, the total number of colonies presented a rising trend during storage at 4 °C and 25 °C. The total number of colonies increased to 3.62 ± 0.22 Log CFU/g at 25 °C on day 8, and increased to Log 3.80 ± 0.19 CFU/g after further storage (day 10), resulting in exceeding the limit level (3.70 Log CFU/g). However, under storage at 4 °C, the total number of colonies reached 3.49 ± 0.14 Log CFU/g at day 18. The SGompertz equation was used for fitting the growth of colonies. The SGompertz growth model parameters were $Y=1.0928\times \exp\left\{-\exp\left[-0.3014\times \left(t-5.4513\right)\right]\right\}$, R^2^ = 0.9947 (25 °C), and $Y=0.8601\times \exp\left\{-\exp\left[-0.1592\times \left(t-12.9969\right)\right]\right\}$ R^2^ = 0.9980 (4 °C). According to the equation, the garlic paste could be stored at 4 °C for 25 days. Therefore, our results indicated that HHP contributed to product preservation.

### 3.6. The Relationship between Color Indexes and Quality-Related Attributes

**Figure 6:**
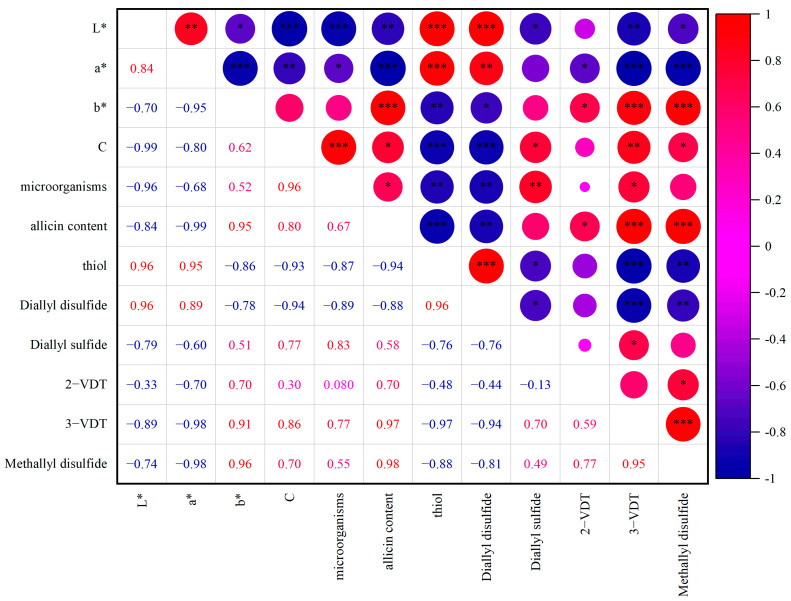
Correlation analysis of color indexes and quality-related attributes. * indicates 0.01 < *p* ≤ 0.05, ** indicates 0.001 < *p* ≤ 0.01, *** indicates *p* ≤ 0.001, respectively.

As shown in Figure 6, the color indexes and quality-related attributes of garlic paste after WPI and HHP treatment were analyzed using Pearson significant correlation analysis. For L* and a*, strong positive correlations were only obtained with DADS and thiol. In addition, it could be seen that allicin showed a strong negative correlation with thiol groups and DADS, while showing a positive correlation with 2-VDT, 3-VDT and methyl disulfide. DADS represented the decomposition level of allicin, and thiol groups were increased with WPI addition and unfolding. From these results, it can be assumed that the control of garlic green discoloration in the presence of WPI is attributed to the exchange of thiol groups with sulfonyl groups in allicin. Moreover, the chroma (C*) index showed a strong positive correlation with allicin content. Allicin played a vital role in green color generation in garlic paste, which was in agreement with previous studies [5,37]. Microorganism counts were positively correlated with methallyl disulfide, which indicated that HPP inactivated microorganisms and decreased the unpleasant odor with WPI unfolding in HPP treatment. Therefore, the WPI-HHP treatment was considered as a potential preservation method, facilitating the inactivation of microorganisms and obtaining a balanced aroma.

## 4. Conclusions

The effects of WPI addition and HHP processing on the microbial inactivation, color parameters, bioactive compounds and volatile aroma profiles of garlic paste, as well as the storage stability, were investigated. Noticeable green discoloration was observed in original garlic paste when HHP processing was employed, corresponding with the changes in L*, a*, b*, and Chroma (C*). In contrast, the addition of WPI to garlic paste (WP) significantly inhibited the garlic’s green discoloration. Further, the application HHP at 500 MPa to WP better preserved the color quality. In consideration of microbial inactivation, color appearance and allicin content of garlic paste, the optimal treatment was as follows: addition of 2% WPI, with HPP parameters of 500 MPa for 5 min. The shelf life of garlic paste with WPI-HHP treatment extended to 25 days. Moreover, GC-MS results revealed that WPI combined with HHP treatment could increase the relative level of the predominant volatile aroma component (diallyl disulfide), while reducing that of unpleasant odor components (methallyl sulfides). It is speculated that WPI reacts with allicin to prevent green discoloration through thiol groups in WPI. The results revealed that WPI combined with HHP treatment show advantages in maintaining garlic paste color quality and the sensory volatile aroma, which provide an important scientific basis for the development of the garlic industry.

## Figures and Tables

**Table 1:** Changes in flavor compounds after WPI and HHP treatment.

Volatile	Relative Abundance (%)			
	GP	WP	HHP-WP	Feature
Diallyl disulfide	19.36	23.50	28.91	Pungency
Diallyl sulfide	1.19	1.13	0.69	Pungency
2-Vinyl-2,4-dihydro-1,3-Dithiin	8.60	5.57	4.17	Garlic scent
3-Vinyl-3,4-dihydro-1,2-dithiin	11.45	7.48	5.96	Garlic scent
Diallyl trisulfide	16.57	15.86	15.25	Pungency
Methallyl disulfide	1.27	0.21	0.19	Unpleasant smell
Diallyl tetrasulfide	0.82	0.67	0.71	Pungency
Methallyl sulfide	2.18	1.22	1.13	Unpleasant smell
2-Vinyl-2,4-dihydro-1,3-dithiin	0.16	0.13	0.14	-
1,3-Ditjiane	0.30	0.53	0.59	-
1,1′-Thiobis-1-propene	5.17	4.32	3.76	-
2-Mercapto-3,4-dimethyl-2,3-dihydrothiophene	0.36	1.00	1.02	-
(Z)-1-(Methylthio)-1-propene	0.25	0.19	0.18	-
2,4-Dimethyl-5,6-dithia-2,7-nonadienal	2.23	1.34	1.05	-
1,3-Benzenedithiol	0.25	0.15	0.13	-
1,2-Dithiolane	0.20	ND	ND	-
1-Propyl-2-(4-thiohept-2-en-5-yl)disulfide	ND	0.13	0.14	-
Cyclotrisiloxane, hexamethyl-	ND	0.89	0.73	-
Methyl allylthioacetate	ND	0.12	0.13	--
Thiophene,2,4-dimethyl-	ND	ND	0.24	-

ND: not detected.

## Data Availability

All related data and methods are presented in this paper. Additional inquiries should be addressed to the corresponding author.

## References

[1] Qiu Z., Zheng Z., Zhang B., Sun-Waterhouse D., Qiao X. (2020). Formation, nutritional value, and enhancement of characteristic components in black garlic: A review for maximizing the goodness to humans. Compr. Rev. Food Sci. Food Saf..

[2] Feng Y., Xu B., Yagoub A.E.A., Ma H., Sun Y., Xu X., Yu X., Zhou C. (2021). Role of drying techniques on physical, rehydration, flavor, bioactive compounds and antioxidant characteristics of garlic. Food Chem..

[3] Putnik P., Gabrić D., Roohinejad S., Barba F.J., Granato D., Mallikarjunan K., Lorenzo J.M., Kovačević D.B. (2019). An overview of organosulfur compounds from *Allium* spp.: From processing and preservation to evaluation of their bioavailability, antimicrobial, and anti-inflammatory properties. Food Chem..

[4] Ramirez D.A., Locatelli D.A., González R.E., Cavagnaro P.F., Camargo A.B. (2017). Analytical methods for bioactive sulfur compounds in *Allium*: An integrated review and future directions. J. Food Compos. Anal..

[5] Kubec R., Curko P., Urajova P., Rubert J., Hajšlová J. (2017). *Allium* discoloration: Color compounds formed during greening of processed garlic. J. Agric. Food Chem..

[6] Qiu Z., Zhang M., Li L., Zhang B., Qiao Y., Zheng Z. (2022). Effect of blend oil on the volatile aroma profile and storage quality of garlic paste. Food Chem..

[7] Zhang B., Qiu Z., Zhao R., Zheng Z., Lu X., Qiao X. (2021). Effect of blanching and freezing on the physical properties, bioactive compounds, and microstructure of garlic (*Allium sativum* L.). J. Food Sci..

[8] Ahmed J., Shivhare U. (2001). Thermal kinetics of color change, rheology, and storage characteristics of garlic puree/paste. J. Food Sci..

[9] Zang J., Wang D., Zhao G. (2013). Mechanism of discoloration in processed garlic and onion. Trends Food Sci. Technol..

[10] Kim K.W., Kim Y.-T., Kim M., Noh B.-S., Choi W.-S. (2014). Effect of high hydrostatic pressure (HHP) treatment on flavor, physicochemical properties and biological functionalities of garlic. LWT-Food Sci. Technol..

[11] Hong S.I., Kim D.M. (2001). Storage quality of chopped garlic as influenced by organic acids and high-pressure treatment. J. Sci. Food Agric..

[12] Lee Y. (2008). Induction of apoptosis by S-allylmercapto-L-cysteine, a biotransformed garlic derivative, on a human gastric cancer cell line. Int. J. Mol. Med..

[13] Liang D., Qin Y., Zhao W., Zhai X., Guo Z., Wang R., Tong L., Lin L., Chen H., Wong Y.-C. (2011). S-allylmercaptocysteine effectively inhibits the proliferation of colorectal cancer cells under in vitro and in vivo conditions. Cancer Lett..

[14] Zhang G., Parkin K.L. (2013). A tissue homogenate method to prepare gram-scale *Allium* thiosulfinates and their disulfide conjugates with cysteine and glutathione. J. Agric. Food Chem..

[15] Jiang H., Xing Z., Wang Y., Zhang Z., Mintah B.K., Dabbour M., Li Y., He R., Huang L., Ma H. (2020). Preparation of allicin-whey protein isolate conjugates: Allicin extraction by water, conjugates’ ultrasound-assisted binding and its stability, solubility and emulsibility analysis. Ultrason. Sonochem..

[16] Huang L., Jia S., Wu R., Chen Y., Ding S., Dai C., He R. (2022). The structure, antioxidant and antibacterial properties of thiol-modified soy protein isolate induced by allicin. Food Chem..

[17] Abd El-Salam M.H., El-Shibiny S. (2018). Glycation of whey proteins: Technological and nutritional implications. Int. J. Biol. Macromol..

[18] Zhang B., Zheng Z., Liu N., Liu P., Qiu Z., Qiao X. (2021). Effect of different combined mechanical and thermal treatments on the quality characteristics of garlic paste. J. Food Sci. Technol..

[19] Tocmo R., Wu Y., Liang D., Fogliano V., Huang D. (2017). Boiling enriches the linear polysulfides and the hydrogen sulfide-releasing activity of garlic. Food Chem..

[20] Ayim I., Ma H., Alenyorege E.A., Ali Z., Donkor P.O., Zhou C. (2018). Integration of ultrasonic treatment in biorefinery of tea residue: Protein structural characteristics and functionality, and the generation of by-products. J. Food Meas. Charact..

[21] Chen X., Chen H., Xiao J., Liu J., Tang N., Zhou A. (2020). Variations of volatile flavour compounds in finger citron (*Citrus medica* L. var. *sarcodactylis*) pickling process revealed by E-nose, HS-SPME-GC-MS and HS-GC-IMS. Food Res. Int..

[22] Gil M.M., Miller F.A., Brandao T.R., Silva C.L. (2011). On the use of the Gompertz model to predict microbial thermal inactivation under isothermal and non-isothermal conditions. Food Eng. Rev..

[23] Zuluaga C., Martínez A., Fernández J., López-Baldó J., Quiles A., Rodrigo D. (2016). Effect of high pressure processing on carotenoid and phenolic compounds, antioxidant capacity, and microbial counts of bee-pollen paste and bee-pollen-based beverage. Innov. Food Sci. Emerg. Technol..

[24] Sun L.-C., Sridhar K., Tsai P.-J., Chou C.-S. (2019). Effect of traditional thermal and high-pressure processing (HPP) methods on the color stability and antioxidant capacities of Djulis (*Chenopodium formosanum* Koidz.). LWT.

[25] Queiroz C., Lopes M.L.M., Da Silva A.J.R., Fialho E., Valente-Mesquita V.L. (2021). Effect of high hydrostatic pressure and storage in fresh-cut cashew apple: Changes in phenolic profile and polyphenol oxidase activity. J. Food Process. Preserv..

[26] Carullo D., Barbosa-Cánovas G., Ferrari G. (2021). Changes of structural and techno-functional properties of high hydrostatic pressure (HHP) treated whey protein isolate over refrigerated storage. LWT.

[27] Zhang Y., Zielinska M., Vidyarthi S.K., Zhao J.-H., Pei Y.-P., Li G., Zheng Z.-A., Wu M., Gao Z.-J., Xiao H.-W. (2020). Pulsed pressure pickling enhances acetic acid transfer, thiosulfinates degradation, color and ultrastructure changes of “Laba” garlic. Innov. Food Sci. Emerg. Technol..

[28] Huang L., Qu L., Jia S., Ding S., Zhao J., Li F. (2022). The interaction of allicin with bovine serum albumin and its influence on the structure of protein. Process Biochem..

[29] Li H., Zhu K., Zhou H., Peng W. (2012). Effects of high hydrostatic pressure treatment on allergenicity and structural properties of soybean protein isolate for infant formula. Food Chem..

[30] Rade-Kukic K., Schmitt C., Rawel H.M. (2011). Formation of conjugates between β-lactoglobulin and allyl isothiocyanate: Effect on protein heat aggregation, foaming and emulsifying properties. Food Hydrocoll..

[31] Ferioli F., Giambanelli E., D’Antuono L.F. (2022). Comparison of Two Extraction Techniques (SDE vs. SPME) for the Determination of Garlic and Elephant Garlic Volatile Compounds. Food Anal. Methods.

[32] Molina-Calle M., Priego-Capote F., de Castro M.D.L. (2017). Headspace−GC–MS volatile profile of black garlic vs fresh garlic: Evolution along fermentation and behavior under heating. LWT.

[33] Ferioli F., Giambanelli E., D’Alessandro V., D’Antuono L.F. (2020). Comparison of two extraction methods (high pressure extraction vs. maceration) for the total and relative amount of hydrophilic and lipophilic organosulfur compounds in garlic cloves and stems. An application to the Italian ecotype “Aglio Rosso di Sulmona”(Sulmona Red Garlic). Food Chem..

[34] Mengers H.G., Schier C., Zimmermann M., Gruhlke M.C., Block E., Blank L.M., Slusarenko A.J. (2022). Seeing the smell of garlic: Detection of gas phase volatiles from crushed garlic (*Allium sativum*), onion (*Allium cepa*), ramsons (*Allium ursinum*) and human garlic breath using SESI-Orbitrap MS. Food Chem..

[35] Momin K., Thomas S. (2020). GC-MS analysis of antioxidant compounds present in different extracts of an endemic plant *Dillenia scabrella* (dilleniaceae) leaves and barks. Int. J. Pharm. Sci. Res.

[36] Auersvald M., Kejla L., Eschenbacher A., Thi H.D., Van Geem K.M., Šimáček P. (2021). Detailed characterization of sulfur compounds in fast pyrolysis bio-oils using GC × GC-SCD and GC–MS. J. Anal. Appl. Pyrolysis.

[37] Zhao R., Zhang B., Sun J., Zheng Z., Qiao X. (2021). Evaluation of degradation of pigments formed during garlic discoloration in different pH. Food Res. Int..

